# Supplementary material for: An Immersible Microgripper for Pancreatic Islet and Organoid Research
Source: Bioengineering (Basel). 2022 Feb 9;9(2):67. doi: 10.3390/bioengineering9020067 (PMC8869445; doi:10.3390/bioengineering9020067)
Supplement: Supplementary file 1 [file bioengineering-09-00067-s001.zip › rums20a2_Supplement-video1.pptx]

## Slide 1
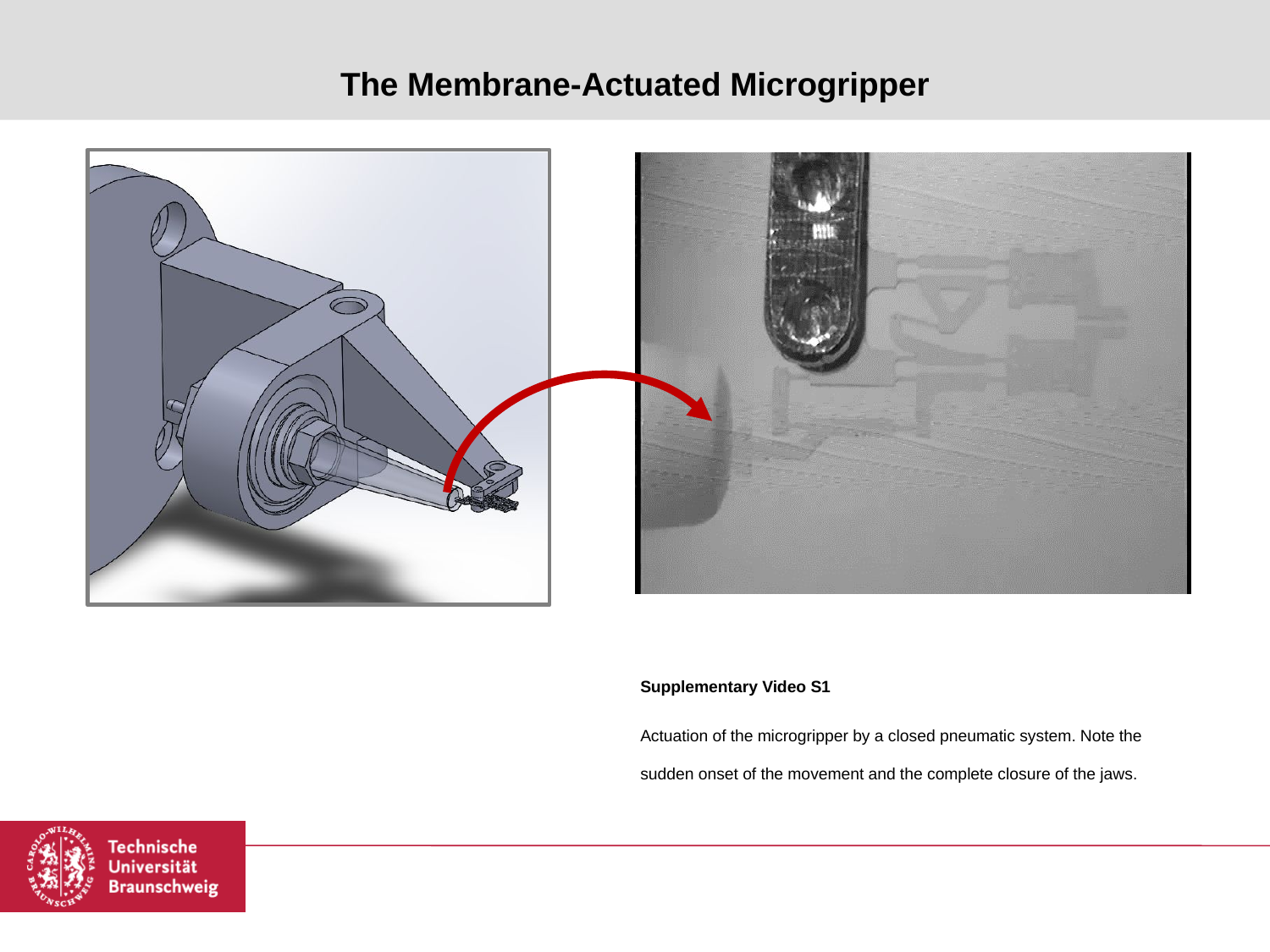

The Membrane-Actuated Microgripper
Supplementary Video S1
Actuation of the microgripper by a closed pneumatic system. Note the sudden onset of the movement and the complete closure of the jaws.
