# Supplementary material for: An Immersible Microgripper for Pancreatic Islet and Organoid Research
Source: Bioengineering (Basel). 2022 Feb 9;9(2):67. doi: 10.3390/bioengineering9020067 (PMC8869445; doi:10.3390/bioengineering9020067)
Supplement: Supplementary file 1 [file bioengineering-09-00067-s001.zip › rums20a2_Supplement-video2.pptx]

## Slide 1
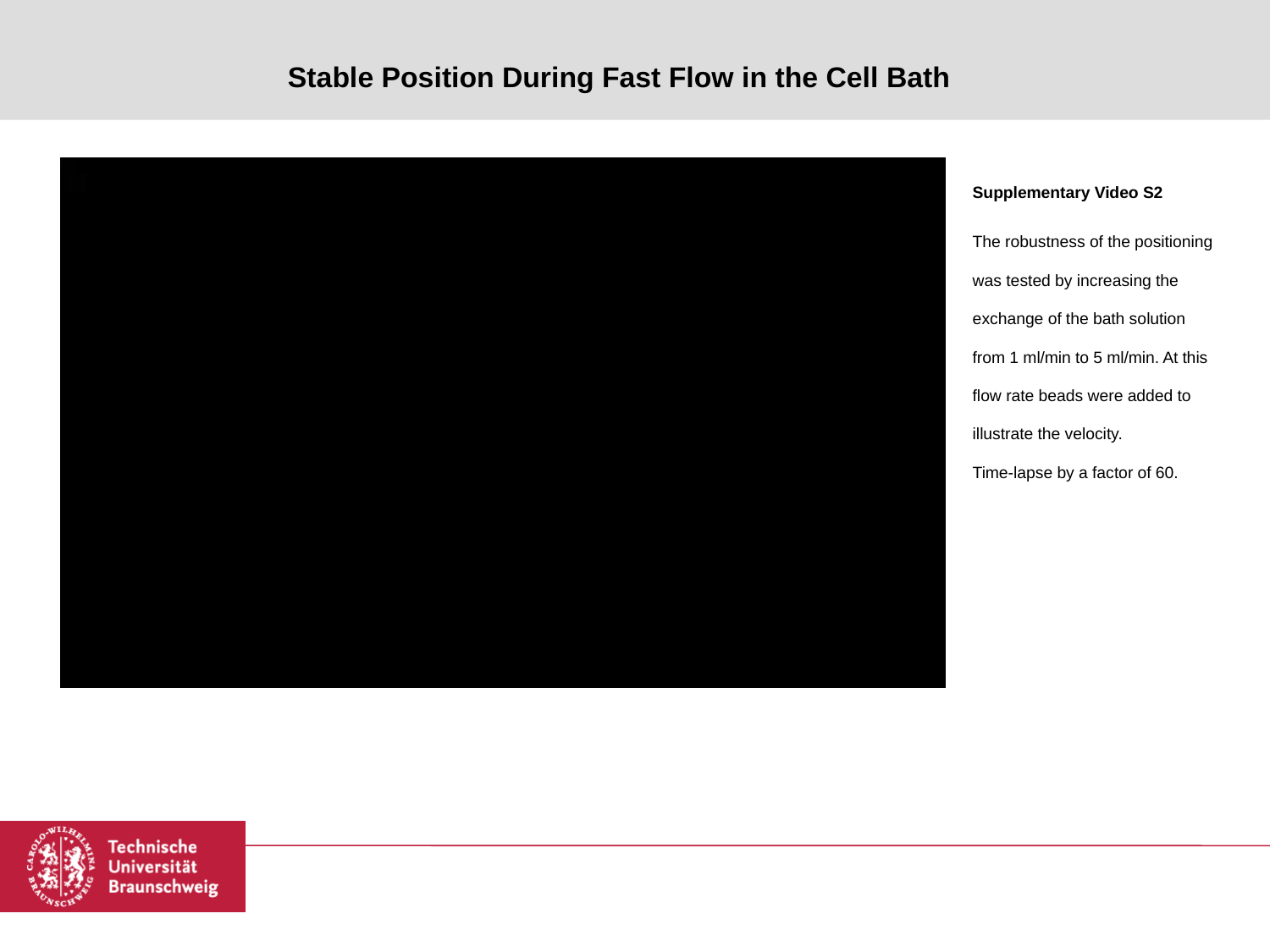

Stable Position During Fast Flow in the Cell Bath
Supplementary Video S2
The robustness of the positioning was tested by increasing the exchange of the bath solution from 1 ml/min to 5 ml/min. At this flow rate beads were added to illustrate the velocity.
Time-lapse by a factor of 60.
